# Did the UN convention on the rights of the child reduce child mortality around the world? An interrupted time series analysis

**DOI:** 10.1186/s12889-020-08720-7

**Published:** 2020-05-18

**Authors:** Christopher A. Tait, Abtin Parnia, Nishan Zewge-Abubaker, Wendy H. Wong, Heather Smith-Cannoy, Arjumand Siddiqi

**Affiliations:** 1 Dalla Lana School of Public Health, University of Toronto, 155 College Street, Toronto, ON M5T 3M7 Canada; 2 Department of Political Science, University of Toronto, 100 St. George Street, Toronto, ON M5S 3G3 Canada; 3 Canada Research Chair, Global Governance and Civil Society; and Schwartz Reisman Research Lead, Toronto, Canada; 4 School of Social and Behavioral Sciences, Arizona State University, 4701 West Thunderbird Rd, MC, Glendale, AZ 3051 USA; 5 Department of Heatlh Behavior, Gillings School of Global Public Health, University of North Carolina – Chapel Hill, 135 Dauer Drive, Chapel Hill, NC 27599 USA

**Keywords:** Child health, Human rights, Global health, United Nations

## Abstract

**Background:**

Child mortality has been reduced by more than 50 % over the past 30 years. A range of secular economic and social developments have been considered to explain this phenomenon. In this paper, we examine the association between ratification of the Convention on the Rights of the Child (CRC), which was specifically put in place to ensure the well-being of children, and declines in child mortality.

**Methods:**

Data come from three sources: the United Nations Treaty Series Database, the World Bank World Development Indicators database and, the Polity IV database. Because CRC was widely ratified, leaving few control cases, we used interrupted times series analyses, which uses the trend in the health outcome before policy exposure to mathematically determine what the trend in the health outcome would have been after the policy exposure, if it had continued ‘as is’ – meaning, if the policy exposure had not occurred.

**Results:**

CRC ratification was associated with declining child mortality. CRC ratification was associated with a significant change in shorter-term child mortality trends in all groups except high-income, non-democratic countries and low-imcome democratic countries. CRC ratification was associated with long-term child mortality trends in all groups except middle-income, non-democratic countries.

**Conclusions:**

Child mortality rates would likely have declined even in the absence of CRC ratification, but CRC is associated with a larger decline. Our findings provide a way to assess the effects of widely-held societal norms on health and demonstrate the moderating effects of democracy and income level.

## Background

According to World Health Organization statistics, child mortality has been reduced by more than 50 % over the past 30 years, from 93 deaths per 1000 live births in 1990 to 41 deaths per 1000 live births in 2016 [1, 2]. At the same time that the benefits have been unequally distributed around the world, the general consensus is that the decline is nonetheless a global phenomenon. Many global changes took place during this period that might offer potential explanations, from poverty reduction to improvements in interventions for diarrhea and other causes of early-life death [3–5]. This analysis focuses on the role of the Convention on the Rights of the Child (CRC), which was adopted and entered into force by the Article 49 of the United Nations General Assembly on September 2, 1990. The CRC was specifically put in place to institutionalize the notion that countries must provide and be accountable for the well-being of children. We are interested in understanding whether declining child mortality rates are associated with the ratification of the CRC by countries around the world.

The CRC followed on earlier twentieth century interest in children’s rights, including the non-binding 1959 Declaration on the Rights of the Child [6]. During the 1980s, high child mortality rates, particularly from highly preventable causes of death, put pressure on donor-receiving countries and lending agencies to take more action. The CRC formally endowed children with a set of rights in the hopes that this would bolster their well-being [7]. It is by far the most adopted of the United Nations’ human rights treaties, ratified by all countries except the United States. As Comstock (2019) has noted, the CRC has had the effect of socializing states in recognizing childrens’ rights, and has significant support from nongovernmental organizations [8]. Although children are protected in other human rights instruments, these were generally viewed as insufficient protection, especially with regard to issues around children’s health [8, 9]. For example, Article 6 of the CRC enshrines the right of every child to life, and the responsibility of every signatory state to “… ensure to the maximum extent possible the survival and development of the child …” [7] Other CRC articles attest to the economic, social, and cultural rights of children, which as the epidemiological and developmental psychology literatures have taught us, are the main inputs, or determinants of survival and development [7].

To date, scarcely anything is known about the extent to which the CRC has actually met one of its core intents; whether it has, in fact, improved the health and well-being of children. The only relevant study we could locate did not explore the association between the CRC and child health, but instead examined whether ratifying more human rights treaties (including the CRC) was associated with improved population health indicators [10]. It was not. Thus, our study investigates the extent to which the CRC itself is associated with improvements in child health, which we measure using a fundamental measure of the children’s well-being, the child mortality rate.

## Methods

### Data sources

Our methodological approach is similar to that of Tait et al. (2019) [11]. We merged three sources of data on country-level characteristics: The United Nations Treaty Series Database, which provides information on treaty ratification, the World Bank World Development Indicators, which provides information on child health and economic conditions and, the Polity IV database, which provides information on political democratization. Supplementary Table 1 provides weblinks to these datasources. Of the 193 countries for which ratification data were available, 192 (99%) had ratified the CRC. As the sole non-ratifying country, the United States was excluded from the sample. The final analytic sample consisted of 192 countries contributing information over the period 1990–2015.

### Measures

Many of our measures have also been previously described in Tait (2019) [11]. Our primary outcome measure was child mortality, which was measured as deaths of children under the age of five per 1000 live births. We wanted to account for a broad range of social, political, and economic factors, which may confound or modify the association between CRC ratification and child mortality rates in a country. However, we were constrained in our ability to use many of the standard approaches for doing so. For example, because almost all countries have ratified the CRC, our sample lacked a control or comparison group of non-ratifying countries that would enable multivariable regression. Because our sample size was relatively small, it precluded conducting an extensive stratified analyses, as we would quickly run out of a sufficient sample size of countries within each stratum of each confounding or moderating variable of interest.

We thus reduced the set of covariates of interest to two variables, which perhaps best comprehensively characterize the conditions of a country. The first was a country’s overall economic conditions at year of CRC ratification, which we measured using per capita gross national income (GNI). GNI represents income acquired through all domestic and foreign sources, which is likely a more accurate reflection of the economic conditions of a country than is gross domestic product (GDP), given today’s more global marketplace. GNI was adjusted for purchasing power parity using the World Bank Atlas Method, which adjusts GNI to reduce changes in the exchange rate that are attributable to inflation. We then divided countries by the World Bank’s income-country groupings: low, lower-middle, upper-middle, and high and, due to sample size constraints, we collapsed the two middle groups into one category. We also examined the split of countries amongst the World health Organizations 6 regions: Africa, Americas, Eastern Mediterranean, Europe, South-East Asia, and Western Pacific.

The second variable represented overall political conditions at the year of CRC ratification for each country, which we measured by the extent to which a country’s political systems were democratized. We measured this using the Polity IV composite index, which ranges from − 10 to 10, with higher values representing greater levels of democratization. Due to sample size constraints, we dichotomized countries between those whose index score was less than 6 (which we termed non-democratic countries) and those whose score was 6 or greater (which we termed democratic countries).

### Statistical analyses

Our analytic strategy is based on that of Tait et al. (2019, 11]. In order to account for differences in the year of ratification, we first standardized the time scale of ratification by ‘zeroing’ at year of ratification for each country. For example, as described in Tait et al. (2019), if a country ratified the CRC in 1980, 1980 was converted to year-zero, and 1985, 5 years post ratification, was converted to year 5 [11].

First, we conducted a series of descriptive statistics to provide a sense of the sample distribution across the variables in our study. Next, we plotted the unadjusted trends in child mortality rates. We then used t-tests to get a sense of the difference between child mortality rates at year of CRC ratification and 5-years and 10-years post ratification. We also used joinpoint regression to determine where there were ‘inflection points’ in child mortality trends; years at which trends in child mortality had changed in ways that are statistically differentiable. Inflection points are fit when the joinpoint regression model determines there is a significant change in the linear trend in the variable (child mortality rate) over time.

These techniques still fall short of testing our central research question, because while they interrogate the trajectory of child mortality rates, they do not tell us enough about how this trajectory may have been affected by CRC ratification. Many gold-standard quasi-experimental methods (e.g. difference-in-differences models) provide strong tests of how a discreet shift in societal conditions, such as a policy change, or indeed ratification of a human rights treaty is associated with an outcome, such as child mortality rates [12]. However, these techniques are reliant on the presence of a ‘control’ group, a group that did not experience the policy or other societal shift, to which we can compare the ‘treatment’ group that was exposed to the shift. The widespread ratification of the CRC (with the exception of the United States) means that there is no control group available to conduct such a model.

Instead, we drew on an alternative quasi-experimental method, interrupted times series analysis (ITSA), which is considered the best alternative in these circumstances [13]. ITSA examines whether the trend in an outcome (child mortality rates) changes after a policy shift (CRC ratification) occurs. ITSA does so by using the pre-shift trend to mathematically construct a projected counterfactual trend: the trend in child mortality, had CRC ratification not occurred. Put differently, it attempts to construct a control group from the treatment group itself. The difference between the actual, observed child mortality trend and the mathematically derived counterfactual trend is the estimated association between CRC ratification and child mortality rates. We analyzed the association with shorter-term trends (5 years post ratification) and longer-term trends (20 years post ratification).

ITSA relies on having multiple observations available in the pre- and post- ratification periods, in order to have a stable indication of trends. However, longer periods of data may then contaminate the analyses by introducing other policy shifts that have influenced trend changes. With this balance in mind, we structured our data to include 5 years of pre-ratification data and up to 20 years of post-ratification data (recent ratifiers contributed less post-ratification data).

Another assumption of ITSA is that the countries in the sample are sufficiently similar, so that we can be fairly confident that any trend changes can be attributable to changes in CRC ratification. In order to account for these confounding and effect-modifying issues, we stratified our ITSA analyses by GNI and democratization. Thus, the country-subgroups in which we conducted separate ITSA analyses were: low-income non-democratic, low-income democratic, middle-income non-democratic, middle-income non-democratic, high-income non-democratic and high-income democratic.

We were also concerned that static measurement of confounders may be problematic and induce misclassification. As Supplementary Table 2 suggests, some, but not many countries moved across democratization categories during the years of data included in the study. Slightly more moved across income groups. An alternative categorization to account for this then might be economic growth rate, rather than economic level itself, since it better characterizes the economic trajectory. However, as Supplemental Table 3 suggests, the set of countries within GNI growth rate categories then becomes even more dissimilar in other respects than when they are categorized by GNI. In other words, the available options are all imperfect, but we believe year of ratification as the basis for categorizing countries by GNI and democratization level is the least problematic of these.

Because there is little prior literature in this area, we are unsure about lag effects. For this reason, and reasons of analytic constraints, we did not test lag effects. All analyses were conducted using Stata/SE version 14 (College Station, TX).

## Results

As demonstrated in Table 1, the CRC ratification period ranged from 1990 to 2015 (median ratification year = 1991). Roughly 25% countries came from the African region, 18% from the Americas, 10% from the Eastern Mediterranean region, 29% from Europe, 6% from South-East Asia, and 12% from the Western Pacific. The vast majority of countries were middle income (*n* = 102) followed by high (*n* = 59) and low-income countries (*n* = 31).

**Table 1:** Characteristics of included countries by UN treaty

Total Countries (n)	192
**Median Ratification Year (range)**	1991 (1990–2015)
**WHO Region (n, %)**	
**Africa**	47 (24.5)
**Americas**	34 (17.7)
**Eastern Mediterranean**	22 (11.5)
**Europe**	54 (28.1)
**South East Asia**	11 (5.7)
**Western Pacific**	24 (12.5)
**Country Income Level**	
**Low**	31 (16.2)
**Middle**	102 (53.1)
**High**	59 (30.7)
**GNI per Capita at Median Ratification Year (mean, PPP Adjusted)**	6236.19

The mean per capita GNI was $6236.19 at the median CRC ratification year. In Supplementary Table 4, we have provided the mean GNI for each income-democraztiation group.

**Fig. 1:**
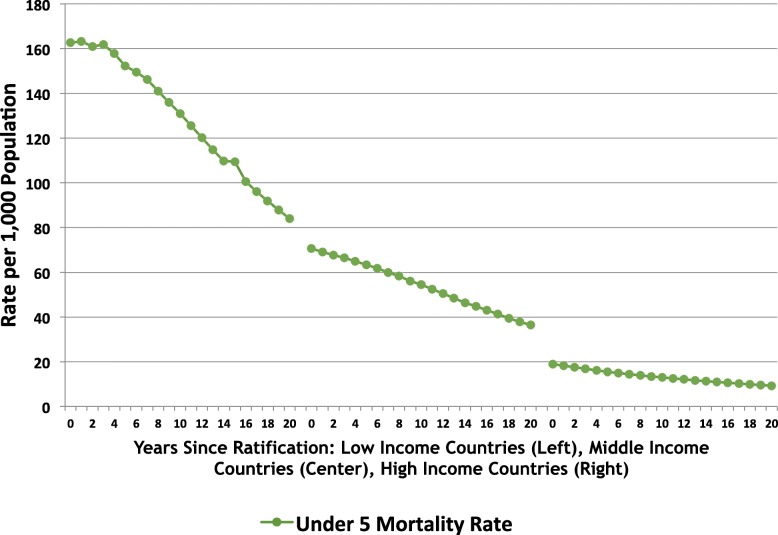
Trends in child mortality post-CRC ratification

In Fig. 1, child mortality rates are observed to have declined over time across countries in all income groups, with the steepest declines occurring in low-income countries. Supplementary Table 5 Provides child mortality rates by country group and year. In Table 2, where these time trends are further interrogated, compared to CRC ratification year, child mortality rates significantly declined 5 years and 10 years after CRC ratification for all country-income groups. At the five-year mark, low-income countries had experienced a decline of 16.4 deaths per 1000 live births, middle-income countries a decline of 8.6 deaths per 1000 live births, and high-income countries a decline of 4.1 deaths per 1000 live births. By the tenth year after ratification, low-income, middle-income, and high-income countries had experienced declines of 37.9, 17.4, and 6.6 deaths per 1000 live births, respectively.

**Table 2:** Child mortality rates pre- vs. post-CRC ratification

	Child Mortality Rate				
	Pre-Ratification	5-Years Post		10-Years Post	
**WHO Region**					
**Africa**	144.4	135.0	*p* = 0.004	119.5	*p* < 0.001
**Americas**	48.0	39.3	p < 0.001	33.2	p < 0.001
**Eastern Mediterranean**	63.8	52.7	p < 0.001	43.6	p < 0.001
**Europe**	24.8	20.4	p < 0.001	16.3	p < 0.001
**South East Asia**	108.7	88.3	*p* = 0.010	69.3	*p* = 0.001
**Western Pacific**	51.8	44.7	p = 0.001	37.3	p = 0.001
**Country Income Level**					
**Low**	168.7	152.3	p = 0.001	130.8	p < 0.001
**Middle**	72.0	63.4	p < 0.001	54.6	p < 0.001
**High**	19.6	15.5	p < 0.001	13.0	p < 0.001

In Table 3, where we examined how trends had changed over time, with no a priori assumptions about when trend changes may occur, depending on the country-category, there were three to four ‘inflection’ points, or distinct changes in the trend (rate of change) in child mortality. For example, amongst low-income, democratic countries, the first three post-ratificaiton years were marked by an 4.8 percentage annual percentage drop in child mortality (95% CI: − 5.0, − 4.6%), but post-ratification years three through eight demonstrated a steeper drop (− 5.7%; 95% CI: − 5.8, − 5.6%), and years eight through fifteen an even steeper drop still (− 6.3%; 95% CI: − 6.8, − 5.8%), with a slight slowing of the rate of decline in years fifteen through twenty. In low-income, non-democratic countries, the frst three post-ratification years had no significant change in child mortality rate, but there were three significant changes in trend over the subsequent 17 years, during which the rate of decline steadily accelerated. The middle income countries (democratic and non-democratic) had steady rates of decline throughout the post-ratification period. In middle-income democratic countries, three trend changes were observed, each in which the rate of decline in child mortality changed by about 1.6%. For middle-income, non-democratic countries, the rate of decline was between 1.3% and 2.1%, also across three trend changes. Finally, the high-income countries demonstrated four distinct trends, but in each, the rate of change was less than 1%. Overall, the average annual percentage across all country groupings ranged from − 0.3% amongst high-income, democratic countries (95% CI, − 0.5, − 0.1%) to − 5.6% in low-income, democratic countries (95% CI, − 6.1, − 5.1%).

**Table 3:** Joinpoint Regression Results, Child Mortality Post-CRC Ratification

	***Mean Under 5 Mortality Rate***		Trend 1		Trend 2		Trend 3		Trend 4		AAPC (95% CI)
	Ratification Year	20 Years Post-Ratification	Year	APC (95% CI)	Year	APC (95% CI)	Year	APC (95% CI)	Year	APC (95% CI)	Overall (0–20 Years)
Low Income, Democratic Countries	208.35	93.96	0–3	−4.8 (−5.0, − 4.6)	3–8	−5.7 (− 5.8, − 5.6)	8–15	− 6.3 (− 6.8, − 5.8)	15–20	− 5.3	− 5.6 (− 6.1, − 5.1)
Low Income, Non-Democratic Countries	158.45	87.96	0–3	1.1 (0.8, 1.4)	3–8	−2.3 (− 2.6, − 2.0)	8–15	−2.2 (− 2.4, − 2.0)	15–20	−4.5	−3.5 (− 3.9, − 3.1)
Middle Income, Democratic Countries	64.99	32.52	0–6	− 1.6 (− 1.9, − 1.3)	6–11	− 1.6 (− 1.7, − 1.5)	11–20	− 1.7 (− 1.7, − 1.7)	–	–	−1.6 (− 1.9, − 1.3)
Middle Income, Non-Democratic Countries	80.36	43.39	0–6	− 1.3 (− 1.3, − 1.3)	6–11	−2.1 (− 2.5, − 1.7)	11–20	− 2.1 (− 2.3, − 1.9)	–	–	−1.8 (− 2.1, − 1.5)
High Income, Democratic Countries	12.41	5.67	0–6	− 0.5 (− 0.5, − 0.4)	6–9	− 0.4 (− 0.5, − 0.3)	9–18	− 0.3 (− 0.5, − 0.1)	18–20	− 0.2 (− 0.2, − 0.2)	−0.3 (− 0.5, − 0.1)
High Income, Non-Democratic Countries	36.25	22.94	0–6	−1.0 (− 1.0, − 1.0)	6–9	− 0.8 (− 0.9, − 0.7)	9–18	−0.8 (− 1.0, − 0.6)	18–20	0.6 (0.6, 0.6)	−0.7 (− 0.1, − 0.6)

(*APC* Annual % Change, *AAPC* Average Annual % Change)

**Fig. 2:**
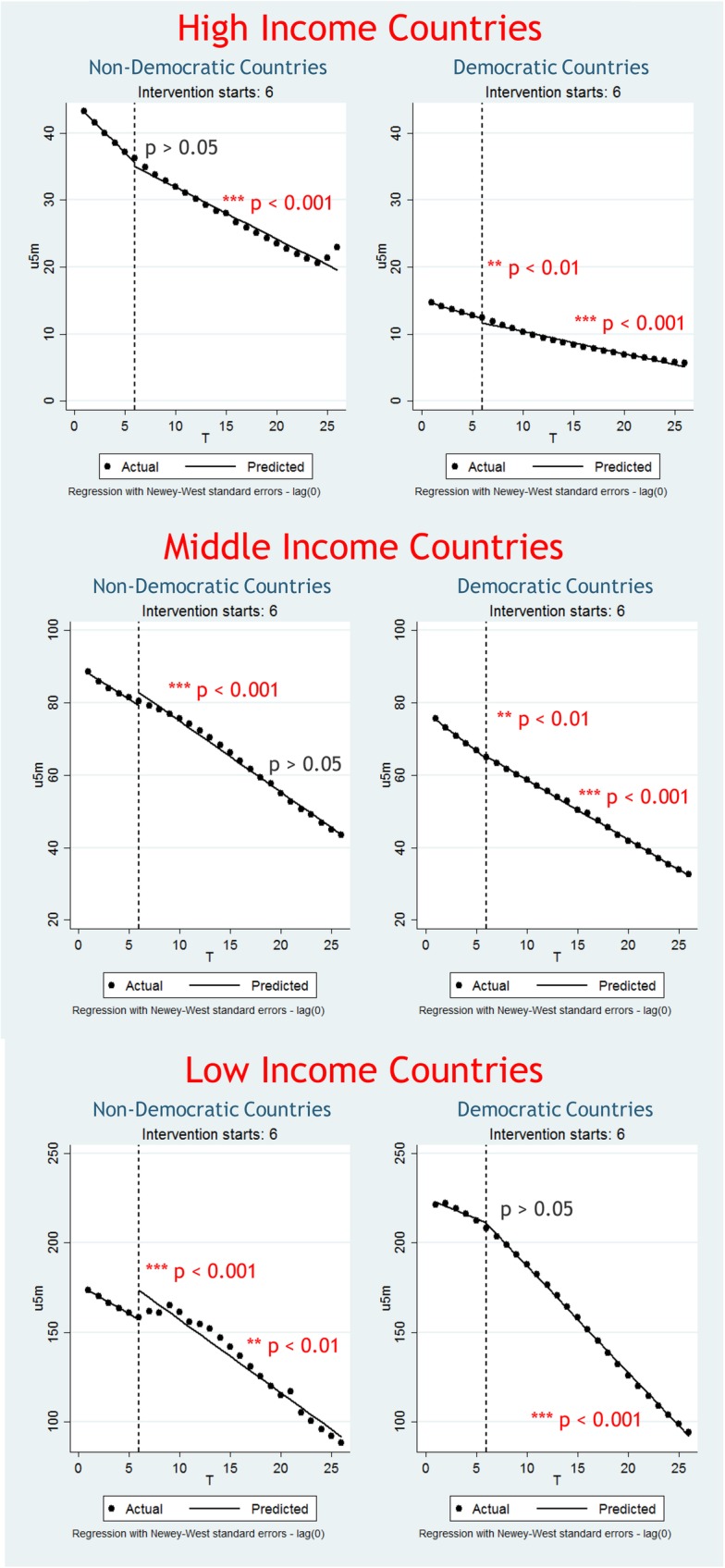
Pre- and post-CRC ratification child mortality rates

Results of the interrupted times series analyses largely indicated that CRC ratification was associated with declining child mortality (Fig. 2). While the graphical representations of the analyses indicated that the actual and ‘counterfactual’ child mortality trends were rather similar, the statistical tests indicated a significant difference between these two scenarios. Among high-income countries, both the short-term and long-term association of CRC ratification with child mortality rates was largely significant, with the exception of the short-term association for high-income, non-democractic countries. Amongst middle-income countries, the association was also widely significant, with the exception of long-term trends in middle-income, non-democratic countries. Among low-imcome countries, only the short-term trend among democratic countries did not yield a significant association.

## Discussion

Our analyses suggest that CRC ratification was broadly associated with a short-term and a long-term decline in child mortality rates. It appears that child mortality rates would likely have declined even in the absence of CRC ratification, but CRC ratification may have facilitated a larger decline. The difference in trends made by ratification ranged from less than five deaths per 1000 population for high-income countries, and less than fifteen deaths per 1000 live births for low-income countries. These are meaningful differences. For high-income countries, the difference is equivalent to the average child mortality rate for 2017. For low-income countries, it is approximately 22% of the 2017 average of 69 deaths per 1000 live births.

We anticipated that greater improvements would have been observed amongst the higher income countries, however, the impact was fairly uniform across income categories. We also anticipated that democracies would demonstrate stronger results. While two of the three non-significant findings were for non-democratic groups (no observable short-term association of CRC ratification with child mortality for high-income, non-democratic countries and no observable long-term association of CRC ratification with child mortality for mid-income countries), it is difficult to say whether this constitutes a true pattern.

While our study suggests an association between ratification of the CRC and reduction in child mortality, it is unclear through which precise mechanisms this association operates. At the outset, we noted that we theorized an association based on the improvements in societal conditions for children that may follow after CRC ratification. Indeed, there is a literature that discusses how societies changed their policy orientations to children by essentially codifying into law that, as a general matter, government actions must be responsive to the CRC [14–16]. There are also indications that specific policies changed as a result of CRC ratification. For instance, in Scotland, the government has made progress towards repealing the legal notion of “justifiable assault” against children [17]. As another example, across many countries, there have been marked investments in early childhood education [16]. Indeed, it seems the CRC is implicated in a rather broad network of living conditions and opportunities that have improved the lives of children, and thus reduced their mortality rates.

Our main study limitation rests in the strength of our control group. When studying the effects of policies and other societal conditions, it is often possible to construct a control group, which has been unaffected by the policy or societal condition but is otherwise similar. In our case, this was not possible due to widespread ratification of the CRC. More substantively, the issue is that it is unclear whether policies and changes in societal conditions that have changed are alternative explanations for reduction in child mortality, or whether they themselves were partially spurred by CRC ratification, or are captured by GNP and democratization. For example, Kuruvilla and colleagues (2014) point out that many countries with the fastest reductions in child mortality had a “cross-sectoral” approach, in which investments were made both within and outside the health sectors [18]. For example, immunizations, and maternal and newborn health care access were important, as were advancements in women’s rights and in general international development [18]. Other studies focus on public health interventions that were both preventive in nature (e.g., breastfeeding) and treatment-oriented (e.g., oral rehydration therapy) [19]. It requires significant further probing beyond the scope of this paper to understand whether and why these interventions are associated with the CRC, or with GNP or democratization.

## Conclusions

Child mortality rates would likely have declined even in the absence of CRC ratification, but CRC is associated with a larger decline.

## Supplementary information

**Additional file 1: Supplementary Table 1.** WEB LOCATIONS FOR DATA SOURCES. **Supplementary Table 2.** Countries that Changed Democratization Categories During the Observation Period. **Supplementary Table 3.** Example of An Alternative Categorization Strategy: GNI Growth Rate. **Supplementary Table 4.** Mean GNI Across All Country Categories. **Supplementary Table 5.** Pre- vs. Post-Ratification Under 5 Mortality Rates by Country Income and Democracy Status.

## Data Availability

The datasets supporting the conclusions of this article are publicly available online. Details regarding navigation instructions are available in Supplementary Table 1. United Nations Treaty Series database is available at: https://treaties.un.org The World Bank World Development Indicators database is available at: http://datatopics.worldbank.org/world-development-indicators/ The Polity IV database is available at: http://www.systemicpeace.org/polityproject.html

## Abbreviations

CRC: Convention on the Rights of the Child
GNI: Gross national income
ITSA: Interrupted-time-series-analysis
