# Supplementary material for: Did the UN convention on the rights of the child reduce child mortality around the world? An interrupted time series analysis
Source: BMC Public Health. 2020 May 18;20:707. doi: 10.1186/s12889-020-08720-7 (PMC7236469; doi:10.1186/s12889-020-08720-7)
Supplement: Supplementary file 1 — Additional file 1: Supplementary Table 1. WEB LOCATIONS FOR DATA SOURCES. Supplementary Table 2. Countries that Changed Democratization Categories During the Observation Period. Supplementary Table 3. Example of An Alternative Categorization Strategy: GNI Growth Rate. Supplementary Table 4. Mean GNI Across All Country Categories. Supplementary Table 5. Pre- vs. Post-Ratification Under 5 Mortality Rates by Country Income and Democracy Status. [file 12889_2020_8720_MOESM1_ESM.docx]

**Supplementary Table 1**

**WEB LOCATIONS FOR DATA SOURCES**

1. **CRC Ratification Year**:

<https://indicators.ohchr.org/>

Navigate to 'Convention on the Rights of the Child', and access each country's ratification year by clicking on the country name in the left-hand panel

1. **WHO Region**:

<https://www.who.int/choice/demography/by_country/en/>

1. **Country income level (Per Capita Gross National Income:**

<https://data.worldbank.org/indicator/NY.GNP.PCAP.PP.CD>

Download Excel file with metadata on all countries over time

1. **Child Mortality Rate**:

<https://data.worldbank.org/indicator/SH.DYN.MORT>

Download Excel file with metadata on all countries over time

1. **Polity IV Index**:

<http://www.systemicpeace.org/inscrdata.html>

Navigate to "Polity IV: Regime Authority Characteristics and Transitions Datasets" and download Excel file with metadata on all countries over time

**Supplementary Table 2**

**Countries that Changed Democratization Categories During the Observation Period**

|  |  |  |  |  |
| --- | --- | --- | --- | --- |
| Country | Ratification Year | Original Democratic Status | Democratic Status Change | Democratic Status Change Year |
|  |  |  |  |  |
|  |  |  |  |  |
| Bangladesh | 1990 | Non-Democratic | Democratic | 1991 |
| Burkina Faso | 1990 | Non-Democratic | Democratic | 2015 |
| Iraq | 1994 | Non-Democratic | Democratic | 2014 |
| Malaysia | 1995 | Non-Democratic | Democratic | 2008 |
| Nigeria | 1991 | Non-Democratic | Democratic | 2015 |
| Thailand | 1992 | Non-Democratic | Democratic | 1993 |

**Supplementary Table 3**

**Example of An Alternative Categorization Strategy: GNI Growth Rate**

| High Income | | Middle Income | | Low Income | |
| --- | --- | --- | --- | --- | --- |
| Below 50th | Above 50th | Below 50th | Above 50th | Below 50th | Above 50th |
| Canada | Israel | Algeria | Algeria | Bangladesh | Benin |
| Sweden | Netherlands | Argentina | Argentina | Kyrgyz Republic | Bolivia |
| Bahrain | Norway | Australia | Australia | Mali | Burkina Faso |
|  | Switzerland | Austria | Austria | Mauritania | India |
|  | Finland | Brazil | Brazil | Pakistan | Papua New Guinea |
|  | Luxembourg | Colombia | Colombia | Philippines | Armenia |
|  | Singapore | Czech Republic | Czech Republic | Uzbekistan | Egypt, Arab Rep. |
|  |  | Dominican Republic | Dominican Republic | Azerbaijan | Eritrea |
|  |  | France | France | Cameroon | Mozambique |
|  |  | Gabon | Gabon | Chad | Rwanda |
|  |  | Jordan | Jordan | Comoros | Sri Lanka |
|  |  | Morocco | Morocco | Kenya | Thailand |
|  |  | South Africa | South Africa | Madagascar |  |
|  |  | Tunisia | Tunisia | Nigeria |  |
|  |  | Ecuador | Belize | Senegal |  |
|  |  | Greece | Bulgaria | Tajikistan |  |
|  |  | Guatemala | Chile | Timor-Leste |  |
|  |  | Honduras | Congo, Rep. | Togo |  |
|  |  | North Macedonia | Cuba | Uganda |  |
|  |  | Peru | Cyprus |  |  |
|  |  | Saudi Arabia | Japan |  |  |
|  |  | Uruguay | Korea, Rep. |  |  |
|  |  | Venezuela, RB | Malaysia |  |  |
|  |  |  | Mauritius |  |  |
|  |  |  | Panama |  |  |
|  |  |  | Seychelles |  |  |
|  |  |  | Syrian Arab Republic |  |  |
|  |  |  | United Kingdom |  |  |

**Supplementary Table 4**

**Mean GNI Across All Country Categories**

|  |  |
| --- | --- |
| Income-Democratization Grouping | Mean GNI |
|  |  |
|  |  |
| Low Income, Democratic | 363 |
| Low Income, Non-Democratic | 269 |
| Middle Income, Democratic | 1,340 |
| Middle Income, Non-Democratic | 1,330 |
| High Income, Democratic | 16,968 |
| High Income, Non-Democratic | 16,202 |

**Supplementary Table 5.** Pre- vs. Post-Ratification Under 5 Mortality Rates by Country Income and Democracy Status

| Low Income - Democratic | |  |  |  | Under 5 Mortality Rates | | |
| --- | --- | --- | --- | --- | --- | --- | --- |
|  | **Country** | **CRC Ratification Year** | **First Year of Observation** | **Last Year of Observation** | **5 Years Pre-Ratification** | **5 Years Post-Ratification** | **10 Years Post-Ratification** |
|  | Benin | 1990 | 1985 | 2015 | 183.9 | 159.1 | 144.7 |
|  | Burundi | 1990 | 1985 | 2015 | 170.7 | 170.5 | 152.2 |
|  | Comoros | 1993 | 1988 | 2015 | 117.2 | 102.7 | 100.4 |
|  | Guinea-Bissau | 1990 | 1985 | 2015 | 233.3 | 205.5 | 177.5 |
|  | Liberia | 1993 | 1988 | 2015 | 255.4 | 205.8 | 145.9 |
|  | Madagascar | 1991 | 1986 | 2015 | 160.7 | 133.4 | 103.1 |
|  | Malawi | 1991 | 1986 | 2015 | 242.4 | 203 | 161.7 |
|  | Mozambique | 1994 | 1989 | 2015 | 227.7 | 180.1 | 140.2 |
|  | Nepal | 1990 | 1985 | 2015 | 147.9 | 107.7 | 80.6 |
|  | Niger | 1990 | 1985 | 2015 | 332.9 | 279.5 | 227.3 |
|  | Sierra Leone | 1990 | 1985 | 2015 | 265.2 | 257.5 | 235.8 |

| Middle Income - Democratic | | |  |  | Under 5 Mortality Rates | | |
| --- | --- | --- | --- | --- | --- | --- | --- |
|  | Country | CRC Ratification Year | First Year of Observation | Last Year of Observation | 5 Years Pre-Ratification | 5 Years Post-Ratification | 10 Years Post-Ratification |
|  | Albania | 1992 | 1987 | 2015 | 38.8 | 30.3 | 23.6 |
|  | Bolivia | 1990 | 1985 | 2015 | 129.2 | 101.8 | 80.2 |
|  | Botswana | 1995 | 1990 | 2015 | 66.1 | 82.9 | 69 |
|  | Brazil | 1990 | 1985 | 2015 | 63.4 | 46.3 | 32 |
|  | Bulgaria | 1991 | 1986 | 2015 | 22.1 | 23.1 | 20.1 |
|  | Cabo Verde | 1992 | 1987 | 2015 | 61 | 49 | 29.9 |
|  | Colombia | 1991 | 1986 | 2015 | 35.1 | 28.9 | 24.3 |
|  | Costa Rica | 1990 | 1985 | 2015 | 17.5 | 15.2 | 13 |
|  | Dominican Republic | 1991 | 1986 | 2015 | 60.2 | 47.3 | 40.1 |
|  | El Salvador | 1990 | 1985 | 2015 | 62.8 | 44.5 | 32.4 |
|  | Georgia | 1994 | 1989 | 2015 | 46 | 37.8 | 26.7 |
|  | Ghana | 1990 | 1985 | 2015 | 132.6 | 113.4 | 100.7 |
|  | Guatemala | 1990 | 1985 | 2015 | 84.9 | 63.3 | 50.6 |
|  | Guyana | 1991 | 1986 | 2015 | 60.4 | 51.4 | 45.6 |
|  | Honduras | 1990 | 1985 | 2015 | 61.1 | 46.4 | 37.4 |
|  | India | 1992 | 1987 | 2015 | 122.2 | 101.9 | 84.3 |
|  | Indonesia | 1990 | 1985 | 2015 | 88.4 | 66.7 | 52.3 |
|  | Jamaica | 1991 | 1986 | 2015 | 30.6 | 25.1 | 21.4 |
|  | Kenya | 1990 | 1985 | 2015 | 99.8 | 114.8 | 107.9 |
|  | Kyrgyz Republic | 1994 | 1989 | 2015 | 64.1 | 51.1 | 40.8 |
|  | Lebanon | 1991 | 1986 | 2015 | 32.5 | 24.6 | 18.8 |
|  | Lesotho | 1992 | 1987 | 2015 | 87.9 | 107.9 | 120.4 |
|  | Macedonia, FYR | 1993 | 1988 | 2015 | 33.5 | 18.1 | 14.2 |
|  | Mauritius | 1990 | 1985 | 2015 | 24.9 | 22.2 | 18.6 |
|  | Mexico | 1990 | 1985 | 2015 | 49 | 35 | 25.6 |
|  | Moldova | 1993 | 1988 | 2015 | 34.2 | 36.3 | 23 |
|  | Mongolia | 1990 | 1985 | 2015 | 113.1 | 84.6 | 62.7 |
|  | Montenegro | 2006 | 2001 | 2015 | 10.4 | 6.2 |  |
|  | Namibia | 1990 | 1985 | 2015 | 75.7 | 70.2 | 75.7 |
|  | Nicaragua | 1990 | 1985 | 2015 | 69.7 | 52.7 | 40.3 |
|  | Pakistan | 1990 | 1985 | 2015 | 141.1 | 125.7 | 112.3 |
|  | Panama | 1990 | 1985 | 2015 | 31.9 | 28.1 | 26 |
|  | Paraguay | 1990 | 1985 | 2015 | 48.2 | 39.2 | 33.5 |
|  | Peru | 1990 | 1985 | 2015 | 84 | 57.5 | 38.6 |
|  | Philippines | 1990 | 1985 | 2015 | 61.7 | 45.6 | 39.7 |
|  | Romania | 1990 | 1985 | 2015 | 39 | 32.3 | 26.9 |
|  | Senegal | 1990 | 1985 | 2015 | 144.1 | 142.1 | 134.9 |
|  | Serbia | 2001 | 1996 | 2015 | 12.6 | 8.4 | 7.3 |
|  | Solomon Islands | 1995 | 1990 | 2015 | 36.2 | 33.1 | 33.2 |
|  | South Africa | 1995 | 1990 | 2015 | 60.1 | 75.3 | 75.2 |
|  | Timor-Leste | 2003 | 1998 | 2015 | 114.4 | 96.4 | 83.3 |
|  | Tunisia | 1992 | 1987 | 2015 | 70.3 | 48.6 | 34.4 |
|  | Turkey | 1995 | 1990 | 2015 | 89.6 | 81.6 | 70.1 |
|  | Zambia | 1991 | 1986 | 2015 | 190.6 | 178.1 | 154.2 |

| High Income - Democratic | |  |  |  | Under 5 Mortality Rates | | |
| --- | --- | --- | --- | --- | --- | --- | --- |
|  | Country | CRC Ratification Year | First Year of Observation | Last Year of Observation | 5 Years Pre-Ratification | 5 Years Post-Ratification | 10 Years Post-Ratification |
|  | Argentina | 1990 | 1985 | 2015 | 28.6 | 23.4 | 20.2 |
|  | Australia | 1990 | 1985 | 2015 | 9.7 | 7 | 6.2 |
|  | Austria | 1992 | 1987 | 2015 | 9 | 6.1 | 5.3 |
|  | Belgium | 1991 | 1986 | 2015 | 10 | 7.1 | 5.6 |
|  | Canada | 1991 | 1986 | 2015 | 8.3 | 6.7 | 6.2 |
|  | Chile | 1990 | 1985 | 2015 | 20.4 | 13.1 | 10.9 |
|  | Croatia | 1992 | 1987 | 2015 | 12.1 | 9.3 | 7.7 |
|  | Cyprus | 1991 | 1986 | 2015 | 11.1 | 8.5 | 6.1 |
|  | Czech Republic | 1993 | 1988 | 2015 | 13.2 | 7.6 | 5.7 |
|  | Denmark | 1991 | 1986 | 2015 | 8.9 | 6.2 | 5.5 |
|  | Estonia | 1991 | 1986 | 2015 | 20.2 | 14.8 | 10.2 |
|  | Finland | 1991 | 1986 | 2015 | 6.7 | 4.9 | 4.2 |
|  | France | 1990 | 1985 | 2015 | 9.3 | 6.5 | 5.4 |
|  | Germany | 1992 | 1987 | 2015 | 8 | 5.9 | 5.1 |
|  | Greece | 1993 | 1988 | 2015 | 11.4 | 8.8 | 6.3 |
|  | Hungary | 1991 | 1986 | 2015 | 19.1 | 13.1 | 10.5 |
|  | Ireland | 1992 | 1987 | 2015 | 8.6 | 7.3 | 6.5 |
|  | Israel | 1991 | 1986 | 2015 | 11.6 | 8.2 | 6.6 |
|  | Italy | 1991 | 1986 | 2015 | 9.7 | 7 | 5.2 |
|  | Japan | 1994 | 1989 | 2015 | 6 | 4.7 | 3.9 |
|  | Korea, Rep. | 1991 | 1986 | 2015 | 7.1 | 5.3 | 6.4 |
|  | Latvia | 1992 | 1987 | 2015 | 22 | 21.6 | 15.1 |
|  | Lithuania | 1992 | 1987 | 2015 | 18 | 13.6 | 10.9 |
|  | Netherlands | 1995 | 1990 | 2015 | 7.1 | 6.2 | 5.4 |
|  | New Zealand | 1993 | 1988 | 2015 | 10 | 7.8 | 6.9 |
|  | Norway | 1991 | 1986 | 2015 | 8.7 | 5.4 | 4.7 |
|  | Poland | 1991 | 1986 | 2015 | 17.3 | 13.3 | 8.8 |
|  | Portugal | 1990 | 1985 | 2015 | 15.9 | 9.7 | 7.2 |
|  | Slovak Republic | 1993 | 1988 | 2015 | 16.3 | 12.7 | 10.6 |
|  | Slovenia | 1992 | 1987 | 2015 | 9.7 | 6.6 | 5 |
|  | Spain | 1990 | 1985 | 2015 | 11.5 | 8.3 | 6.5 |
|  | Sweden | 1990 | 1985 | 2015 | 8.4 | 6.4 | 5.6 |
|  | Switzerland | 1997 | 1992 | 2015 | 28.1 | 21.4 | 17.3 |
|  | Trinidad and Tobago | 1991 | 1986 | 2015 | 57 | 42.1 | 29.6 |
|  | United Kingdom | 1991 | 1986 | 2015 | 23.1 | 20 | 16.2 |
|  | Uruguay | 1990 | 1985 | 2015 | 72.5 | 69.5 | 63.2 |

| Low Income - Non-Democratic |  |  |  | Under 5 Mortality Rates | | |
| --- | --- | --- | --- | --- | --- | --- |
| Country | CRC Ratification Year | First Year of Observation | Last Year of Observation | 5 Years Pre-Ratification | 5 Years Post-Ratification | 10 Years Post-Ratification |
| Afghanistan | 1994 | 1989 | 2015 | 162 | 139.9 | 123.2 |
| Burkina Faso | 1990 | 1985 | 2015 | 203.4 | 199.4 | 185.7 |
| Cambodia | 1992 | 1987 | 2015 | 117.4 | 122.5 | 88.3 |
| Central African Republic | 1992 | 1987 | 2015 | 176.2 | 176.9 | 171.9 |
| Chad | 1990 | 1985 | 2015 | 217.3 | 202.7 | 190.2 |
| Congo, Dem. Rep. | 1990 | 1985 | 2015 | 188.5 | 176.4 | 161 |
| Eritrea | 1994 | 1989 | 2015 | 131.2 | 94 | 72.9 |
| Ethiopia | 1991 | 1986 | 2015 | 204.6 | 168.3 | 139 |
| Gambia, The | 1990 | 1985 | 2015 | 176.6 | 142.4 | 118.8 |
| Guinea | 1990 | 1985 | 2015 | 243.7 | 205.9 | 170.2 |
| Haiti | 1995 | 1990 | 2015 | 129.1 | 104.8 | 90 |
| Korea, Dem. Rep. | 1990 | 1985 | 2015 | 39.5 | 72.8 | 60 |
| Mali | 1990 | 1985 | 2015 | 259.5 | 240 | 219.6 |
| Rwanda | 1991 | 1986 | 2015 | 151.8 | 203.3 | 170.2 |
| Somalia | 2015 | 2015 | 2015 | 141.2 |  |  |
| South Sudan | 2015 | 2015 | 2015 | 95.6 |  |  |
| Tanzania | 1991 | 1986 | 2015 | 37 | 27 | 21.5 |
| Togo | 1990 | 1985 | 2015 | 22.7 | 18.9 | 17.6 |
| Uganda | 1990 | 1985 | 2015 | 19.8 | 20.5 | 18.5 |
| Zimbabwe | 1990 | 1985 | 2015 | 73.9 | 95.5 | 105.8 |

| Middle Income - Non-Democratic |  |  |  | Under 5 Mortality Rates | | |
| --- | --- | --- | --- | --- | --- | --- |
| Country | CRC Ratification Year | First Year of Observation | Last Year of Observation | 5 Years Pre-Ratification | 5 Years Post-Ratification | 10 Years Post-Ratification |
| Algeria | 1993 | 1988 | 2015 | 44.9 | 40.6 | 36.5 |
| Angola | 1990 | 1985 | 2015 | 226.2 | 224.8 | 216.7 |
| Armenia | 1993 | 1988 | 2015 | 45.2 | 33.3 | 25.9 |
| Azerbaijan | 1992 | 1987 | 2015 | 95 | 88.6 | 63.8 |
| Bangladesh | 1990 | 1985 | 2015 | 149.7 | 114.2 | 88 |
| Belarus | 1990 | 1985 | 2015 | 17 | 17.9 | 14.3 |
| Bhutan | 1990 | 1985 | 2015 | 139.8 | 105.3 | 79.6 |
| Bosnia and Herzegovina | 1993 | 1988 | 2015 | 18.4 | 10.7 | 8.2 |
| Cameroon | 1993 | 1988 | 2015 | 142.8 | 155.3 | 134.7 |
| China | 1992 | 1987 | 2015 | 53.3 | 43.6 | 31.6 |
| Congo, Rep. | 1993 | 1988 | 2015 | 97.4 | 119.3 | 109.8 |
| Cote d'Ivoire | 1991 | 1986 | 2015 | 152.6 | 152.2 | 142.8 |
| Cuba | 1991 | 1986 | 2015 | 13.3 | 10.5 | 8 |
| Djibouti | 1990 | 1985 | 2015 | 122 | 110.2 | 100.8 |
| Ecuador | 1990 | 1985 | 2015 | 59.7 | 43.9 | 34.4 |
| Egypt, Arab Rep. | 1990 | 1985 | 2015 | 90.6 | 64.6 | 46.5 |
| Fiji | 1993 | 1988 | 2015 | 28 | 25.1 | 23.7 |
| Gabon | 1994 | 1989 | 2015 | 90.6 | 86.3 | 78.8 |
| Iran, Islamic Rep. | 1994 | 1989 | 2015 | 49.6 | 36.8 | 27.3 |
| Iraq | 1994 | 1989 | 2015 | 51 | 45.6 | 41.5 |
| Jordan | 1991 | 1986 | 2015 | 36.6 | 30.7 | 26.9 |
| Kazakhstan | 1994 | 1989 | 2015 | 52.9 | 45.9 | 34.7 |
| Lao PDR | 1991 | 1986 | 2015 | 162.4 | 135.5 | 113.4 |
| Libya | 1993 | 1988 | 2015 | 37.6 | 29.9 | 25.5 |
| Malaysia | 1995 | 1990 | 2015 | 13.9 | 10.2 | 8.2 |
| Mauritania | 1991 | 1986 | 2015 | 118.1 | 113.9 | 113.1 |
| Morocco | 1993 | 1988 | 2015 | 73 | 54.8 | 43.9 |
| Myanmar | 1991 | 1986 | 2015 | 109.9 | 92.7 | 79.9 |
| Nigeria | 1991 | 1986 | 2015 | 212.5 | 205.1 | 181.3 |
| Papua New Guinea | 1993 | 1988 | 2015 | 86.6 | 80.2 | 76.1 |
| Sri Lanka | 1991 | 1986 | 2015 | 21.2 | 19.5 | 15.8 |
| Sudan | 1990 | 1985 | 2015 | 48.9 | 40.9 | 34.4 |
| Suriname | 1993 | 1988 | 2015 | 78.8 | 117.7 | 133.7 |
| Swaziland | 1995 | 1990 | 2015 | 5.2 | 4.1 | 3.6 |
| Syrian Arab Republic | 1993 | 1988 | 2015 | 115.7 | 105.6 | 74.6 |
| Tajikistan | 1993 | 1988 | 2015 | 162.6 | 144.5 | 106.9 |
| Thailand | 1992 | 1987 | 2015 | 168.2 | 127.8 | 99.1 |
| Turkmenistan | 1993 | 1988 | 2015 | 55.4 | 46 | 38.7 |
| Ukraine | 1991 | 1986 | 2015 | 16.5 | 12.6 | 10.9 |
| Uzbekistan | 1994 | 1989 | 2015 | 31.9 | 28.8 | 27.9 |
| Vietnam | 1990 | 1985 | 2015 | 46.7 | 35.7 | 29.8 |
| Yemen, Rep. | 1991 | 1986 | 2015 | 126.3 | 109.8 | 90.7 |

| High Income - Non-Democratic |  |  |  | Under 5 Mortality Rates | | |
| --- | --- | --- | --- | --- | --- | --- |
| Country | CRC Ratification Year | First Year of Observation | Last Year of Observation | 5 Years Pre-Ratification | 5 Years Post-Ratification | 10 Years Post-Ratification |
| Bahrain | 1992 | 1987 | 2015 | 22.7 | 15.2 | 11.8 |
| Equatorial Guinea | 1992 | 1987 | 2015 | 185.8 | 163.9 | 143.8 |
| Kuwait | 1991 | 1986 | 2015 | 17.9 | 14.2 | 12.5 |
| Oman | 1996 | 1991 | 2015 | 24.5 | 15.5 | 12.3 |
| Qatar | 1995 | 1990 | 2015 | 16.1 | 12.4 | 10.3 |
| Russian Federation | 1990 | 1985 | 2015 | 26.4 | 26.4 | 23.2 |
| Saudi Arabia | 1996 | 1991 | 2015 | 29.7 | 22 | 19.2 |
| Singapore | 1995 | 1990 | 2015 | 5.4 | 4 | 2.9 |
| United Arab Emirates | 1997 | 1992 | 2015 | 7 | 6.3 | 5.8 |
| Venezuela, RB | 1990 | 1985 | 2015 | 52.8 | 41.2 | 33.8 |
